# Crystal structure of poly[bis­(μ-2-bromo­pyrazine)­tetra-μ_2_-cyanido-dicopper(I)iron(II)]: a bimetallic metal-organic framework

**DOI:** 10.1107/S2056989018016638

**Published:** 2018-11-30

**Authors:** Olesia I. Kucheriv, Inna I. Tokmenko, Igor P. Matushko, Galyna G. Tsapyuk, Il’ya A. Gural’skiy

**Affiliations:** a Department of Chemistry, Taras Shevchenko National University of Kyiv, Volodymyrska St 64, Kyiv 01601, Ukraine; b UkrOrgSyntez Ltd, Chervonotkatska St 67, Kyiv 02094, Ukraine

**Keywords:** crystal structure, bromo­pyrazine, di­cyano­cuprate, iron(II), copper(I), bimetallic, metal-organic framework, MOF

## Abstract

In the title metal-organic framework, [Fe(C_4_H_3_BrN_2_)_2_{Cu(CN)_2_}_2_]_*n*_, the Fe^II^ cation is located on an inversion center and has a slightly elongated octa­hedral coordination environment [FeN_6_], ligated by two pyrazine N atoms of symmetry-related bridging 2-bromo­pyrazine mol­ecules in the axial positions and by four N atoms of pairs of symmetry-related cyanido groups in the equatorial positions. The Cu^I^ center has a fourfold coordination environment [CuC_3_N], with an almost perfect trigonal–pyramidal geometry, formed by three cyanido C atoms and an N atom of a bridging 2-bromo­pyrazine mol­ecule. Copper(I) centers related by a twofold rotation axis are bridged by two carbon atoms from a pair of μ-CN groups, resulting in Cu_2_(CN)_2_ units. Each Cu_2_(CN)_2_ unit is linked to six Fe^II^ cations via a pair of linear CN units, the pair of μ-CN groups and two bridging 2-bromo­pyrazine ligands, resulting in the formation of a metal–organic framework, which is additionally stabilized by the short Cu⋯Cu contacts of 2.4450 (7) Å.

## Abstract 2

In the title bimetallic metal–organic framework, {Fe(Brpz)_2_[Cu(CN)_2_]_2_}_*n*_, where Brpz is 2-bromo­pyrazine, the Fe^II^ cation is located on an inversion centre and has a slightly elongated octa­hedral FeN_6_ coordination environment. The Cu^I^ center has a fourfold CuC_3_N coordination environment with an almost perfect trigonal–pyramidal geometry. Copper(I) centers related by a twofold rotation axis are bridged by two carbon atoms from a pair of μ-CN groups, resulting in Cu_2_(CN)_2_ units that build up the coordination framework.

## Chemical context   

The rational design and synthesis of cyanide-based coordination materials is of key inter­est today. By using different approaches for their design and tunable structures, composition and porosity, various exciting properties of these compounds, such as catalytic, photoluminescent, magnetic, electrical and other can be achieved (Zhang *et al.*, 2015 ▸; Catala & Mallah, 2017 ▸). The cyanide anion is an important ligand in coordination chemistry as it can be used for stabilization of coordination materials formed by diverse transition metals. Cyanide-containing coordination materials of very different topologies have been proposed, although attention is frequently paid to heterometallic complexes.
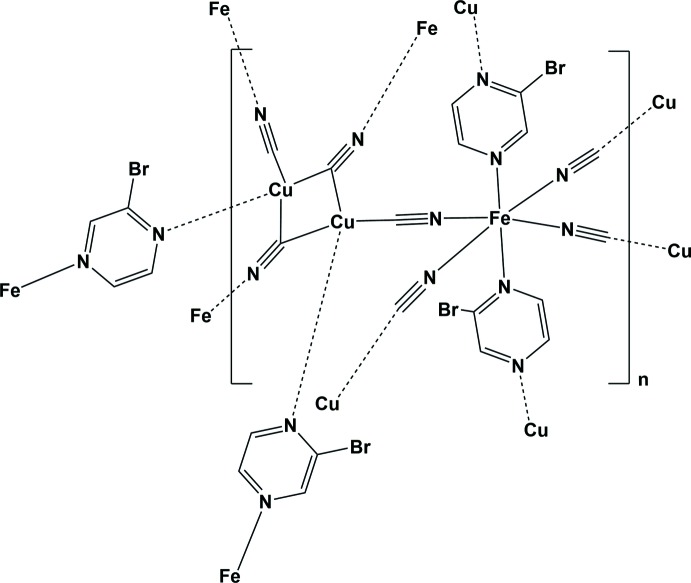


The first class of cyanide-based metallic complexes emerged in the 18th century with the discovery of Prussian blue. Later its analogues containing two types of metals were synthesized. The inclusion of different metals instead of iron resulted in the occurrence of various attractive physical properties of these materials, which allowed their use as mol­ecular sieves, for nanoscale devices, for hydrogen storage, *etc*. (Newton *et al.*, 2011 ▸). Prussian blue analogues form networks with general formula *AM*_A_[*M_B_*(CN)_6_] (*A* = alkali metal ion, *M_A_* and *M_B_* = transition metal ions) (Keggin & Miles, 1936 ▸). These complexes have a cubic structure in which the metallic centers are bridged in an *M_A_*—C≡N—*M_B_* fashion, forming three-dimensional frameworks.

Another class of heterometallic cyanide coordination compounds that has attracted much attention is represented by *f*–*d* complexes composed of lanthanide(III) ions and *d*-block cyano­metallates. This type of materials has been shown to have exceptional photoluminescent properties (Chorazy *et al.*, 2017 ▸). In addition, polynuclear octa­cyanides form a different family of cyanide-based heterometallic complexes. Compounds of this class can adopt very different geometries creating 0, 1, 2 or 3D assemblies. These materials are known for photomagnetism, mol­ecular magnetism, and the ability to create chiral networks (Sieklucka *et al.*, 2011 ▸).

The Hofmann clathrate analogues of the general formula [*M_A_*(*L*)_*x*_{*M_B_*(CN)_*y*_}] constitute another prominent example of bimetallic cyano­metallates (Hofmann & Küspert, 1897 ▸), which are famous for their switchable magnetic properties (Muñoz & Real, 2011 ▸). Here we describe the crystal structure of a new Hofmann clathrate analogue of general formula [Fe(Brpz)_2_{Cu(CN)_2_}_2_]_*n*_.

## Structural commentary   

**Figure 1:**
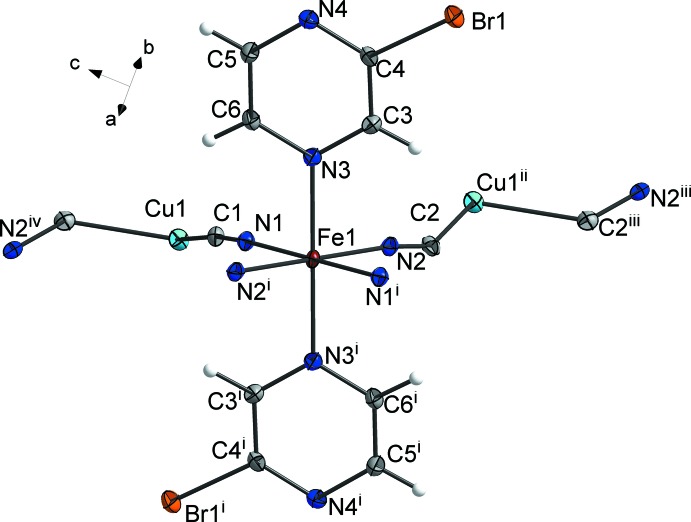
A fragment of the crystal structure of the title compound, with atom labelling. Displacement ellipsoids are drawn at 50% probability level [symmetry codes: (i) −*x* + 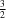, −*y* + 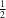, −*z* + 1; (ii) −*x* + 1, −*y*, −*z* + 1; (iii) −*x* + 1, *y*, −*z* + 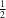; (iv) *x*, −*y*, *z* + 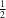].

A fragment of the structure of the title compound, illustrating the sixfold coordination environment of atom Fe1, is shown in Fig. 1 ▸. Selected geometrical parameters are given in Table 1 ▸. The Fe^II^ ion is located on an inversion centre and has a slightly elongated FeN_6_ octa­hedral coordination environment. It is ligated by two N atoms of symmetry-related 2-bromo­pyrazine mol­ecules in axial positions [Fe1—N3 = 1.980 (2) Å] and by four N atoms of symmetry related cyanido groups in the equatorial positions [Fe1—N1 = 1.958 (2) and Fe1—N2 = 1.952 (2) Å]. The Fe—N bond lengths clearly indicate that the Fe^II^ center is in the low-spin state at the temperature of the experiment, *i.e.* 296 K. The deviation from an ideal octa­hedron of the Fe^II^ center can be described by the octa­hedral distortion parameter Σ|90 − θ| = 23.36°, where θ is a *cis*-N—Fe—N angle. Notably, this sum is higher than that expected for a low-spin Fe^II^ ion. It is important to note, and should always be taken into account, that the octa­hedral distortion value cannot always be used to judge the spin state of a metallic center, it is rather a characteristic of the order level in the structure, the measurement temperature, *etc*.

**Figure 2:**
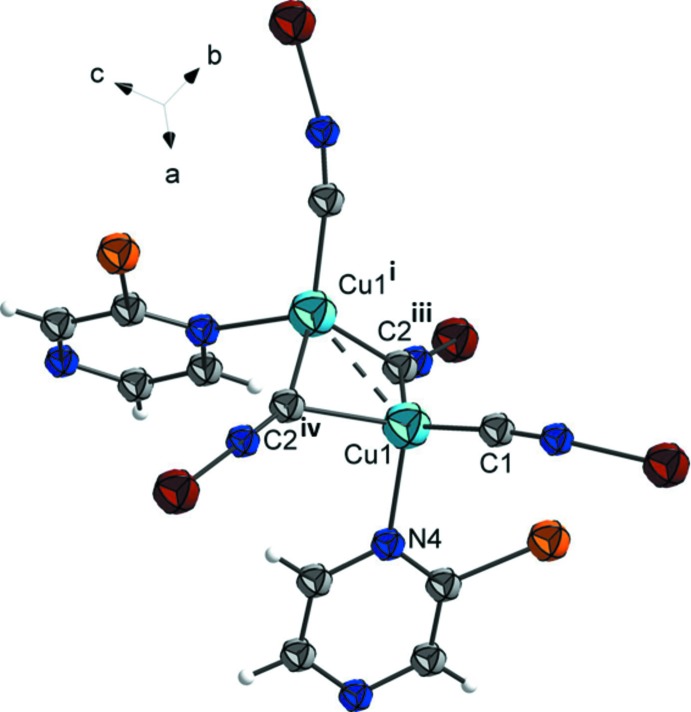
A view of the coordination environment of the Cu atoms in the title compound, with atom labelling [symmetry codes: (i) −*x* + 1, −*y*, −*z* + 1; (iii) *x*, −*y*, *z* + 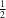; (iv) *x*, −*y*, *z* + 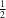].

Atom Cu1 has a fourfold CuC_3_N coordination environment (Fig. 2 ▸, Table 1 ▸) with a τ_4_ descriptor of 0.82, close to that for a perfect trigonal–pyramidal geometry (τ_4_ = 0.00 for square-planar, 1.00 for tetra­hedral and 0.85 for trigonal–pyramidal; Yang *et al.*, 2007 ▸). It is ligated by three C atoms of the cyanido groups and an N atom of a bridging 2-bromo­pyrazine mol­ecule [Cu1—C1 = 1.924 (3), Cu1—C2^iv^ = 2.181 (3), Cu1—C2^iii^ = 2.049 (3), Cu1—N4^ii^ = 2.152 (2) Å]. Notably, the coord­ination to atom Fe1 occurs only via the more sterically accessible atom N3 of the pyrazine ring, while atom Cu1 binds to atom N4 of the pyrazine ring.

## Supra­molecular features   

**Figure 3:**
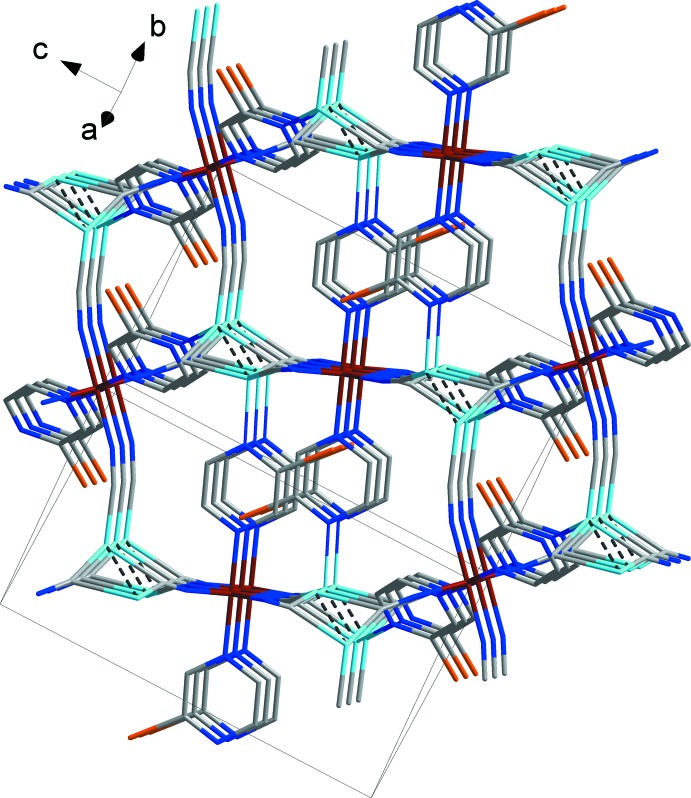
A view normal to plane (110) of the crystal structure of the title compound, showing the Cu⋯Cu contacts as dashed lines. H atoms have been omitted for clarity. Colour code: Fe dark red, Cu light blue, C grey, N blue, Br orange.

The crystal packing of the title compound is shown in Fig. 3 ▸. The coordination framework is made up of bridging 2-bromo­pyrazine ligands and Cu_2_(CN)_2_ moieties (Fig. 2 ▸). The latter are formed by a pair of copper atoms, centered about a twofold rotation axis, being bridged by two carbon atoms from a pair of μ-CN groups. Each Cu_2_(CN)_2_ unit is linked to six Fe^II^ cations via a pair of linear CN units, the pair of μ-CN groups and two bridging 2-bromo­pyrazine ligands, resulting in the formation of a metal–organic framework (Fig. 3 ▸). The framework is additionally stabilized by the short Cu1⋯Cu1^i^ contact of 2.4550 (7) Å, which is significantly shorter than the sum of the corresponding van der Waals radii, *viz*. 2.8 Å. Additionally, within the framework there are weak Br⋯π contacts of 3.8298 (6) Å, that are of lone-pair⋯π origin.

## Database survey   

A search of the Cambridge Structural Database (CSD, version 5.39, last update August 2018; Groom *et al.*, 2016 ▸) gave 15 hits for Fe—N≡C—Cu bimetallic structures supported mainly by substituted pyridines and pyrimidines. These include a number of variable temperature measurements of certain compounds in order to study their spin-crossover behaviour; for example, the two-dimensional coordination polymer catena-[bis­[(μ^3^-cyano-*C*,*C*,*N*)(μ^2^-cyano-*C*,*N*)]tetra­kis­(3-cyano­pyrid­yl)dicop­per(I)iron(II)] (VEHHOG, VEHHOG01; Galet *et al.*, 2006 ▸), and the framework structures *catena*-[tetra­kis­(μ^2^-cyano-*C*,*N*)bis­(μ^2^-pyrimidine-*N*,*N*′)dicopper(I)iron(II)] (EHOQOH01, EHOQOH03; Agustí *et al.*, 2008 ▸) and *catena*-[octa­kis­(μ^2^-cyano)­octa­kis­(3-chloro­pyridine)­tetra­copper(I)diiron(II)] (YUBRET, YUBRET03; Agustí *et al.*, 2009 ▸).

A search of the CSD for the bridging Cu_2_(CN)_2_ unit gave 27 hits. The majority of these are monometallic copper(I) metal–organic frameworks (MOFs). The Cu⋯Cu distances vary from *ca* 2.31 Å in the two-dimensional network structure *catena*-[bis­(μ_3_-cyano)­tetra­kis­(μ_2_-cyano)­tris­(*N*,*N*,*N*′,*N*′-tetra­methyl­ethylenedi­amine)­hexa­copper(I)] (HIWHUQ; Stocker *et al.*, 1999 ▸) to *ca* 2.72 Å in the MOF *catena*-[tris­(μ-cyano)­tris­(μ-cyano)­diammine­penta­copper] (OPODAA; Grifasi *et al.*, 2016 ▸). Two particular compounds resemble the title MOF, namely *catena*-[bis­(μ_3_-cyano-*C*,*C*,*N*)(μ_2_-cyano-*C*,*N*)(μ_2_-2,3-di­methyl­pyrazine-*N*,*N*′)tricopper(I)] (FERWUV; Greve & Nather, 2004 ▸), which involves a bridging 2,3-di­methyl­pyrazine ligand, and *catena*-[(μ_3_-cyano)(μ_2_-4,4′-bi­pyridine)­tris­(μ_2_-cyano)­hexa­methyl­dicopper(I)ditin(IV)] (NUMRUI; Ibrahim *et al.*, 1998 ▸), which is a bimetallic MOF with a bridging 4,4′-bi­pyridine ligand. The respective Cu⋯Cu distances are *ca* 2.49 and 2.62 Å, compared to 2.4550 (7) Å in the title MOF.

## Synthesis and crystallization   

Crystals of the title compound were obtained by slow diffusion within three layers in a 3 ml glass tube. The first layer was a solution of K[Cu(CN)_2_] (7.8 mg, 0.05 mmol) in 1 ml of water; the second layer was a water/ethanol mixture (1:1, 1 ml); the third layer was a solution of Fe(ClO_4_)_2_·6H_2_O (9.1 mg, 0.025 mmol) and 2-bromo­pyrazine (8.0 mg, 0.05 mmol) in 0.5 ml of ethanol. After two weeks, brown crystals were formed in the middle layer. The crystals were kept under the mother solution prior to measurement.

## Refinement   

Crystal data, data collection and structure refinement details are summarized in Table 2 ▸. All the hydrogen atoms were placed geometrically and refined as riding: C—H = 0.93 Å with U_iso_(H) = 1.2U_eq_(C).

## Supplementary Material

Crystal structure: contains datablock(s) I, Global. DOI: 10.1107/S2056989018016638/su5461sup1.cif

Structure factors: contains datablock(s) I. DOI: 10.1107/S2056989018016638/su5461Isup2.hkl

CCDC reference: 1880717

Additional supporting information:  crystallographic information; 3D view; checkCIF report

## Figures and Tables

**Table 1:** Selected geometric parameters (Å, °)

Cu1—Cu1^i^	2.4450 (7)	Cu1—C2^iv^	2.181 (3)
Cu1—N4^ii^	2.152 (2)	Fe1—N1	1.958 (2)
Cu1—C1	1.924 (3)	Fe1—N2	1.952 (2)
Cu1—C2^iii^	2.049 (3)	Fe1—N3	1.980 (2)
			
N4^ii^—Cu1—C2^iv^	99.77 (10)	N3—Fe1—N3^v^	180
C2^iii^—Cu1—N4^ii^	94.29 (10)	N2^v^—Fe1—N3	87.27 (9)
C2^iii^—Cu1—C2^iv^	104.00 (10)	N2—Fe1—N3	92.73 (9)
C1—Cu1—N4^ii^	109.09 (10)	N2^v^—Fe1—N1	92.19 (9)
C1—Cu1—C2^iv^	116.07 (11)	N2—Fe1—N1	87.81 (9)
C1—Cu1—C2^iii^	128.08 (11)	N1—Fe1—N3	90.92 (9)
N1—Fe1—N1^v^	180	N1^v^—Fe1—N3	89.08 (9)
N2—Fe1—N2^v^	180		

Symmetry codes: (i) 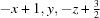; (ii) 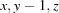; (iii) 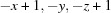; (iv) 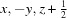; (v) 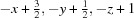.

**Table 2:** Experimental details

Crystal data	
Chemical formula	[Cu_2_Fe(CN)_4_(C_4_H_3_BrN_2_)_2_]
*M*_r_	605.00
Crystal system, space group	Monoclinic, *C*2/*c*
Temperature (K)	296
*a*, *b*, *c* (Å)	13.6143 (17), 9.3067 (11), 13.2101 (15)
β (°)	92.369 (4)
*V* (Å^3^)	1672.3 (3)
*Z*	4
Radiation type	Mo *K*α
μ (mm^−1^)	8.17
Crystal size (mm)	0.2 × 0.1 × 0.05

Data collection	
Diffractometer	Bruker SMART
Absorption correction	Multi-scan (*SADABS*; Bruker, 2013 ▸)
*T*_min_, *T*_max_	0.487, 0.746
No. of measured, independent and observed [*I* > 2σ(*I*)] reflections	8711, 2023, 1799
*R*_int_	0.029
(sin θ/λ)_max_ (Å^−1^)	0.661

Refinement	
*R*[*F* ^2^ > 2σ(*F* ^2^)], *wR*(*F* ^2^), *S*	0.026, 0.063, 1.04
No. of reflections	2023
No. of parameters	115
H-atom treatment	H-atom parameters constrained
Δρ_max_, Δρ_min_ (e Å^−3^)	1.08, −0.76

Computer programs: *APEX2* and *SAINT* (Bruker, 2013 ▸), *SHELXS* (Sheldrick, 2008 ▸), *SHELXL* (Sheldrick, 2015 ▸), *OLEX2* (Dolomanov *et al.*, 2009 ▸) and *DIAMOND* (Brandenburg, 1999 ▸).

## supplementary crystallographic information

### Crystal data

**Table:**

[Cu_2_Fe(CN)_4_(C_4_H_3_BrN_2_)_2_]	*F*(000) = 1152
*M**_r_* = 605.00	*D*_x_ = 2.403 Mg m^−^^3^
Monoclinic, *C*2/*c*	Mo *K*α radiation, λ = 0.71073 Å
*a* = 13.6143 (17) Å	Cell parameters from 3458 reflections
*b* = 9.3067 (11) Å	θ = 2.7–28.0°
*c* = 13.2101 (15) Å	µ = 8.17 mm^−^^1^
β = 92.369 (4)°	*T* = 296 K
*V* = 1672.3 (3) Å^3^	Plate, brown
*Z* = 4	0.2 × 0.1 × 0.05 mm

### Data collection

**Table:**

Bruker SMART diffractometer	1799 reflections with *I* > 2σ(*I*)
φ and ω scans	*R*_int_ = 0.029
Absorption correction: multi-scan (SADABS; Bruker, 2013)	θ_max_ = 28.0°, θ_min_ = 2.7°
*T*_min_ = 0.487, *T*_max_ = 0.746	*h* = −17→17
8711 measured reflections	*k* = −12→12
2023 independent reflections	*l* = −17→17

### Refinement

**Table:**

Refinement on *F*^2^	Primary atom site location: structure-invariant direct methods
Least-squares matrix: full	Secondary atom site location: difference Fourier map
*R*[*F*^2^ > 2σ(*F*^2^)] = 0.026	Hydrogen site location: inferred from neighbouring sites
*wR*(*F*^2^) = 0.063	H-atom parameters constrained
*S* = 1.04	*w* = 1/[σ^2^(*F*_o_^2^) + (0.0308*P*)^2^ + 4.9236*P*] where *P* = (*F*_o_^2^ + 2*F*_c_^2^)/3
2023 reflections	(Δ/σ)_max_ = 0.001
115 parameters	Δρ_max_ = 1.08 e Å^−^^3^
0 restraints	Δρ_min_ = −0.76 e Å^−^^3^

### Special details

**Table:**

Geometry. All esds (except the esd in the dihedral angle between two l.s. planes) are estimated using the full covariance matrix. The cell esds are taken into account individually in the estimation of esds in distances, angles and torsion angles; correlations between esds in cell parameters are only used when they are defined by crystal symmetry. An approximate (isotropic) treatment of cell esds is used for estimating esds involving l.s. planes.

### Fractional atomic coordinates and isotropic or equivalent isotropic displacement parameters (Å^2^)

**Table:**

	*x*	*y*	*z*	*U*_iso_*/*U*_eq_	
Br1	0.58637 (3)	0.80466 (3)	0.43290 (2)	0.02782 (10)	
Cu1	0.56339 (2)	−0.11583 (4)	0.68710 (2)	0.01446 (10)	
Fe1	0.750000	0.250000	0.500000	0.00911 (12)	
N2	0.64165 (17)	0.2082 (2)	0.40313 (17)	0.0125 (4)	
N4	0.61897 (18)	0.6878 (3)	0.62526 (18)	0.0167 (5)	
N3	0.68414 (17)	0.4233 (2)	0.55166 (16)	0.0131 (4)	
N1	0.68406 (17)	0.1288 (2)	0.59751 (17)	0.0133 (4)	
C6	0.6791 (2)	0.4482 (3)	0.6517 (2)	0.0179 (6)	
H6	0.697279	0.376181	0.697551	0.022*	
C2	0.5807 (2)	0.1643 (3)	0.3484 (2)	0.0144 (5)	
C3	0.6541 (2)	0.5292 (3)	0.4890 (2)	0.0167 (5)	
H3	0.654572	0.515097	0.419324	0.020*	
C5	0.6474 (2)	0.5785 (3)	0.6873 (2)	0.0205 (6)	
H5	0.645476	0.591957	0.756995	0.025*	
C1	0.6393 (2)	0.0431 (3)	0.63961 (19)	0.0129 (5)	
C4	0.6224 (2)	0.6594 (3)	0.5265 (2)	0.0164 (5)	

### Atomic displacement parameters (Å^2^)

**Table:**

	*U*^11^	*U*^22^	*U*^33^	*U*^12^	*U*^13^	*U*^23^
Br1	0.0441 (2)	0.02245 (17)	0.01682 (15)	0.01597 (14)	0.00049 (13)	0.00483 (11)
Cu1	0.01237 (17)	0.01466 (17)	0.01645 (17)	−0.00143 (13)	0.00172 (13)	0.00342 (13)
Fe1	0.0097 (3)	0.0095 (2)	0.0081 (2)	−0.00185 (19)	−0.00025 (19)	0.00029 (18)
N2	0.0139 (12)	0.0124 (10)	0.0114 (10)	0.0000 (9)	0.0031 (9)	0.0019 (8)
N4	0.0199 (13)	0.0148 (11)	0.0154 (11)	0.0042 (9)	0.0019 (10)	0.0007 (9)
N3	0.0124 (11)	0.0152 (11)	0.0118 (10)	−0.0003 (9)	0.0006 (9)	−0.0006 (9)
N1	0.0133 (11)	0.0142 (11)	0.0122 (10)	0.0002 (9)	−0.0011 (9)	−0.0018 (9)
C6	0.0245 (16)	0.0162 (13)	0.0130 (12)	0.0054 (11)	−0.0003 (11)	0.0026 (10)
C2	0.0162 (14)	0.0109 (11)	0.0158 (12)	0.0015 (10)	−0.0011 (10)	0.0019 (10)
C3	0.0193 (14)	0.0181 (13)	0.0126 (12)	0.0047 (11)	0.0005 (10)	−0.0022 (10)
C5	0.0302 (17)	0.0198 (14)	0.0115 (12)	0.0071 (12)	0.0019 (11)	0.0004 (11)
C1	0.0142 (13)	0.0144 (13)	0.0101 (11)	0.0008 (10)	−0.0001 (10)	0.0014 (9)
C4	0.0166 (14)	0.0173 (13)	0.0151 (13)	0.0054 (11)	−0.0003 (10)	0.0041 (10)

### Geometric parameters (Å, º)

**Table:**

Br1—C4	1.884 (3)	N2—C2	1.153 (4)
Cu1—Cu1^i^	2.4450 (7)	N4—C5	1.353 (4)
Cu1—N4^ii^	2.152 (2)	N4—C4	1.334 (4)
Cu1—C1	1.924 (3)	N3—C6	1.347 (3)
Cu1—C2^iii^	2.049 (3)	N3—C3	1.340 (4)
Cu1—C2^iv^	2.181 (3)	N1—C1	1.160 (4)
Fe1—N1	1.958 (2)	C6—H6	0.9300
Fe1—N2^v^	1.952 (2)	C6—C5	1.376 (4)
Fe1—N2	1.952 (2)	C3—H3	0.9300
Fe1—N3	1.980 (2)	C3—C4	1.385 (4)
Fe1—N3^v^	1.980 (2)	C5—H5	0.9300
Fe1—N1^v^	1.958 (2)		
			
N4^ii^—Cu1—Cu1^i^	121.81 (7)	C2—N2—Fe1	170.7 (2)
N4^ii^—Cu1—C2^iv^	99.77 (10)	C5—N4—Cu1^vi^	120.31 (19)
C2^iii^—Cu1—Cu1^i^	57.25 (8)	C4—N4—Cu1^vi^	124.53 (19)
C2^iv^—Cu1—Cu1^i^	52.20 (8)	C4—N4—C5	115.0 (2)
C2^iii^—Cu1—N4^ii^	94.29 (10)	C6—N3—Fe1	121.39 (19)
C2^iii^—Cu1—C2^iv^	104.00 (10)	C3—N3—Fe1	121.14 (18)
C1—Cu1—Cu1^i^	128.71 (8)	C3—N3—C6	116.9 (2)
C1—Cu1—N4^ii^	109.09 (10)	C1—N1—Fe1	167.4 (2)
C1—Cu1—C2^iv^	116.07 (11)	N3—C6—H6	119.4
C1—Cu1—C2^iii^	128.08 (11)	N3—C6—C5	121.1 (3)
N1—Fe1—N1^v^	180	C5—C6—H6	119.4
N2—Fe1—N2^v^	180	Cu1^iii^—C2—Cu1^vii^	70.56 (9)
N3—Fe1—N3^v^	180	N2—C2—Cu1^iii^	151.5 (2)
N2^v^—Fe1—N3	87.27 (9)	N2—C2—Cu1^vii^	137.1 (2)
N2^v^—Fe1—N3^v^	92.73 (9)	N3—C3—H3	119.5
N2—Fe1—N3	92.73 (9)	N3—C3—C4	120.9 (3)
N2—Fe1—N3^v^	87.27 (9)	C4—C3—H3	119.5
N2^v^—Fe1—N1	92.19 (9)	N4—C5—C6	122.7 (3)
N2—Fe1—N1	87.81 (9)	N4—C5—H5	118.6
N2—Fe1—N1^v^	92.19 (9)	C6—C5—H5	118.6
N2^v^—Fe1—N1^v^	87.81 (9)	N1—C1—Cu1	170.1 (2)
N1—Fe1—N3	90.92 (9)	N4—C4—Br1	118.8 (2)
N1^v^—Fe1—N3^v^	90.92 (9)	N4—C4—C3	123.2 (3)
N1^v^—Fe1—N3	89.08 (9)	C3—C4—Br1	118.0 (2)
N1—Fe1—N3^v^	89.08 (9)		
			
Cu1^vi^—N4—C5—C6	176.8 (2)	N3—C3—C4—N4	−0.5 (5)
Cu1^vi^—N4—C4—Br1	5.2 (3)	C6—N3—C3—C4	2.0 (4)
Cu1^vi^—N4—C4—C3	−176.7 (2)	C3—N3—C6—C5	−2.0 (4)
Fe1—N3—C6—C5	169.6 (2)	C5—N4—C4—Br1	−179.1 (2)
Fe1—N3—C3—C4	−169.7 (2)	C5—N4—C4—C3	−1.0 (4)
N3—C6—C5—N4	0.6 (5)	C4—N4—C5—C6	1.0 (5)
N3—C3—C4—Br1	177.6 (2)		

Symmetry codes: (i) −*x*+1, *y*, −*z*+3/2; (ii) *x*, *y*−1, *z*; (iii) −*x*+1, −*y*, −*z*+1; (iv) *x*, −*y*, *z*+1/2; (v) −*x*+3/2, −*y*+1/2, −*z*+1; (vi) *x*, *y*+1, *z*; (vii) *x*, −*y*, *z*−1/2.
